# Resistance training's impact on blood biomarkers and cognitive function in older adults with low and high risk of mild cognitive impairment: a randomized controlled trial

**DOI:** 10.1186/s11556-024-00344-9

**Published:** 2024-04-10

**Authors:** Wouter A. J. Vints, Evrim Gökçe, Julija Šeikinaitė, Simona Kušleikienė, Vida J. Česnaitienė, Jeanine Verbunt, Oron Levin, Nerijus Masiulis

**Affiliations:** 1 Department of Health Promotion and Rehabilitation, Lithuanian Sports University, Sporto Str. 6, 44221 Kaunas, Lithuania; 2 Department of Rehabilitation Medicine Research School CAPHRI, Maastricht University, Maastricht, The Netherlands; 3 Centre of Expertise in Rehabilitation and Audiology, Adelante Zorggroep, Hoensbroek, The Netherlands; 4 Sports Rehabilitation Laboratory, Ankara City Hospital, 06800 Ankara, Turkey; 5 Department of Rehabilitation, Physical and Sports Medicine, Institute of Health Science, Vilnius University, Vilnius, Lithuania; 6 Department of Imaging and Pathology, Group Biomedical Sciences, Biomedical MRI Unit, Catholic University Leuven, Leuven, Belgium

**Keywords:** Cognition, Resistance training, Aging, Mild cognitive impairment, Inflammation, Neurotrophic factor, Myokine, Exercise, RCT

## Abstract

**Background:**

The aging brain exhibits a neuroinflammatory state, driven partly by peripheral pro-inflammatory stimuli, that accelerates cognitive deterioration. A growing body of evidence clearly indicates that physical exercise partly alleviates neuroinflammation and positively affects the aging process and cognition. In this randomized controlled trial, we aimed to observe the effect of 12 weeks of resistance training (RT) on peripheral biomarker levels, cognitive function changes and their interrelationship, and explore differences in those exercise-induced changes in older adults with high risk of mild cognitive impairment (MCI) compared to older adults with low risk of MCI.

**Methods:**

Fifty-two participants ﻿(aged 60–85 years old, 28 female) were randomly allocated to a 12 week lower limb RT program consisting of two training sessions per week or waiting list control group. The Montreal Cognitive Assessment (MoCA) was used to stratify participants screened as high (< 26/30) or low risk (≥ 26/30) of MCI. We assessed serum Interleukin 6 (IL-6), Insulin-like Growth Factor-1 (IGF-1), and Kynurenine (KYN) levels. Cognitive measurement consisted of and four subtests of Automated Neuropsychological Assessment Metrics (ANAM), the two-choice reaction time, go/no-go, mathematical processing, and memory search test.

**Results:**

Twelve weeks of RT improved Go/No-go test results in older adults with high MCI risk. RT did not significantly affect blood biomarkers. However, IGF-1 level increases were associated with improvements in response time on the mathematical processing test in the exercise group, and IL-6 level increases were associated with improvements in response time on the memory search test in the total group of participants. Finally, KYN levels significantly differed between older adults with low and high MCI risk but no significant associations with performance were found.

**Conclusion:**

Our study results suggest a different effect of RT on inhibitory control between older adults with low compared to high MCI risk. IGF-1 may play a role in the mechanism behind the cognitive benefit of RT and KYN may be a surrogate biomarker for neurodegeneration and cognitive decline.

**Supplementary Information:**

The online version contains supplementary material available at 10.1186/s11556-024-00344-9.

## Introduction

Cognitive decline is a natural part of aging and can have a significant impact on an individual's quality of life and ability to live independently [1]. When objective evidence of cognitive impairment is present, the terminology employed distinguishes between mild cognitive impairment (MCI) and dementia, with the latter encompassing more than one cognitive domain and being characterized by a substantial interference with an individual's daily life [2]. MCI, considered a preclinical, but still reversible, stage between healthy aging and dementia, is viewed as a potential target for interventions aiming to delay progression towards dementia [3, 4]. The worldwide prevalence of MCI is substantial, manifesting in 15.6% of community-dwelling adults aged 50 years and older [4]. Moreover, the worldwide prevalence of dementia (57.4 million in 2019) is continuously rising, even at a faster pace than can solely be explained by the gradual increase in older adults living in our society, and expected to almost triple by 2050 [5–7]. Therefore, it is argued that interventions targeting risk factors of dementia and factors that are known to affect reversal from MCI to healthy aging, such as physical exercise, need to be implemented [3, 6]. However, the complexity of the underlying mechanisms and the heterogeneity of potential approaches makes that researchers are still unable to compose the optimal exercise treatment strategy.

Evidence from systematic reviews and meta-analyses consistently demonstrated that regular exercise improves cognitive function in older adults [8, 9]. However, the effect of exercise on cognition is subject to variation based on the exercise modalities employed, the cognitive domains selected, as well as the participant’s cognitive status. For instance, a network meta-analysis on the effect of exercise to improve cognition in older adults indicated that resistance training (RT) appears to have larger beneficial effects on cognitive and motor functioning than other exercise modalities, although more research has been done on aerobic exercise training [10]. In general, the beneficial effect of exercise was found for all subcognitive domains, with resistance exercise having the greatest benefits on executive function, according to a meta-analysis [11]. Another meta-analysis in individuals with MCI showed that RT improved cognition and alleviated MCI [12]. Additionally, a meta-analysis on the effect of aerobic exercise indicated a larger effect size for improvements in cognition in participants with MCI compared to healthy and demented participants [13]. To the best of our knowledge, no similar analysis exists for RT interventions.

While the exact mechanism for this effect is not fully understood, some studies have suggested a role of exercise-induced anti-inflammatory and neurotrophic blood biomarkers which could serve as precursors for exercise-induced neuroplasticity [14, 15]. On the one hand, older adults with higher levels of circulating inflammatory markers and lower levels of neurotrophic factors have been found to have a higher risk of cognitive decline and the development of neurodegenerative diseases [16–18]. On the other hand, there is a considerable body of literature showing that physical activity can reduce the expression of pro-inflammatory markers, such as interleukin-6 (IL-6) and kynurenine (KYN), and increase levels of neurotrophic factors, such as insulin-like growth factor-1 (IGF-1) in both cognitively intact older adults and older individuals with neurodegenerative disease conditions [19–21].

Based on these considerations, the primary aim of this exploratory study was to examine the relationship between RT-induced changes in blood levels of IL-6, KYN and IGF-1, and changes in cognitive function (specifically, processing speed and executive functions) in older adults and investigate whether this relationship was affected by the cognitive health status (high versus low MCI risk) of the older adult. First, we expected that resistance exercise would increase serum IGF-1 and would decrease serum IL-6 and KYN levels (hypothesis 1). Second, we expected that resistance exercise would improve cognitive performance (hypothesis 2). Based on the two previous hypotheses we further hypothesized that resistance exercise-induced changes in blood biomarkers and cognitive performance would be interrelated (hypothesis 3). Lastly, we expected that the changes induced by resistance exercise would be larger in older adults with a high MCI risk than in cognitively healthy ones (hypothesis 4).

## Methods

### Ethical approval and participants

Based on a priori sample size calculation done in G*Power 3.1, we needed to include 52 participants in order to find an interaction effect with medium effect size using a repeated measures ANOVA test with alpha 0.05 and power 0.80. Taking into account the possibility of drop-outs, seventy older adults were included in the study. Participants were eligible for the study if they: (1) were 60 years and older; (2) were not currently on psychopharmacological medication or had used these types of drugs in the last five years; (3) voluntarily participated in the study; (4) were fluent in Lithuanian language; (5) were not regularly participating in any exercise program during the previous six months.

Exclusion criteria were: (1) musculoskeletal disorders, especially of the lower extremity hindering participation in the exercise group; (2) neurological disorders such as previous brain injuries, stroke, multiple sclerosis, epilepsy, or neurodegenerative diseases, or a Montreal Cognitive Assessment (MoCA) score below 16/30 indicating possible undiagnosed dementia [22]; (3) psychiatric disorders such as depression or alcohol or drug abuse in the last five years; (4) diabetes mellitus; (5) deep vein thrombosis; (6) oncologic diseases or history of chemotherapy use; or participants that were not allowed or able to undergo magnetic resonance imaging (MRI) based on the exclusion checklist provided by the Department of Radiology, Lithuanian University of Health Science. MR data collected in the study are not presented in this article. For MRI results, see Vints et al., 2022, 2023 [23, 24]; Sheoran et al., 2023 [25]; Valatkeviciene et al., 2023 [26]; and Levin et al., 2023 [27].

Participants were recruited and continuously enrolled between July 2020 and July 2021 via presentations in local community organizations and contacting candidates from a list of patients provided by general practitioners. Interested individuals were invited to Saules Family Medical Centre, where the study's goals, objectives, and methodology were explained in detail. Participants gave written consent prior to study enrolment. The protocol was approved by the Kaunas Regional Biomedical Research Ethics Committee (No. BE-10–7). All participants signed an informed consent form prior to their inclusion in the study.

### Study design

We conducted a single-blinded, two-arm randomized controlled trial with a 12 weeks intervention with lower body resistance exercises at the Institute of Sports Science and Innovation, Lithuanian Sports University. Randomization was performed using a stratified 8-blocked randomization process, stratifying by MoCA score (below 26/30 and 26–30/30), so that each block contained two participants with low MoCA score (i.e., MoCA < 26) and two participants with high MoCA score (i.e., MoCA ≥ 26) allocated to the control group, and two participants with low MoCA score and two participants with high MoCA score allocated to the experimental group. The random allocation was accomplished in an Excel spreadsheet using a random number generator set to indicate either 1 or 2 for the exercise or control group. If two participants from the same group were in a block (N) of four participants with the same cognitive status, a third participant with the same cognitive status and allocated to the same group was assigned to block N + 1. The final block of eight participants included only participants at high risk of MCI, and seven were assigned to the control group. We took this decision to correct for the higher number of drop-outs in the control group participants with high risk of MCI at the beginning of the project. The reason for the difference in number of drop-outs most likely existed because participants allocated to the control group were less motivated to return for follow-up assessments. Assessors of the outcome measurements were blinded for the allocation of the participants to the experimental or control group. The participants were not blind to their group allocation.

**Fig. 1:**
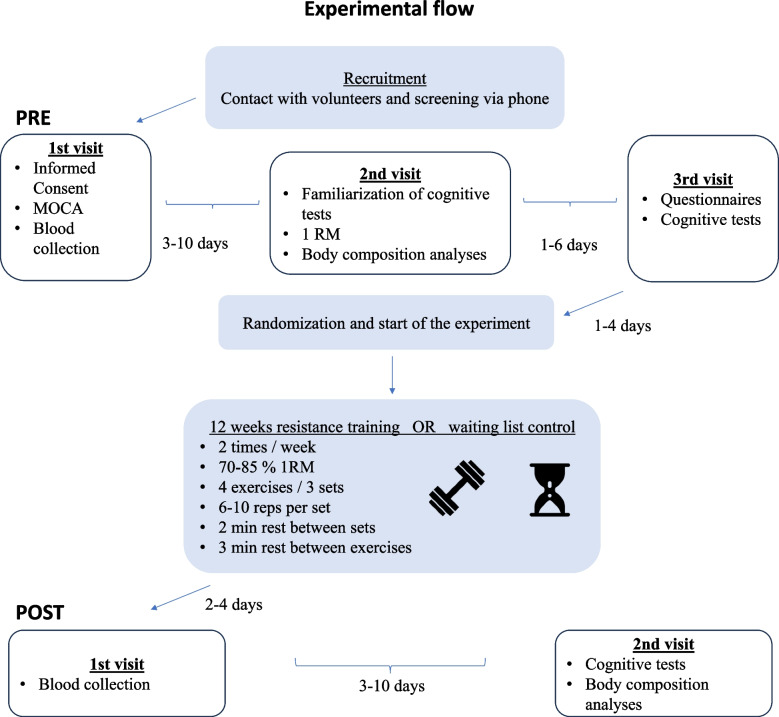
Experimental flow chart. Abbreviations: MOCA, Montreal Cognitive Assessment; RM, repetition maximum

**Fig. 2:**
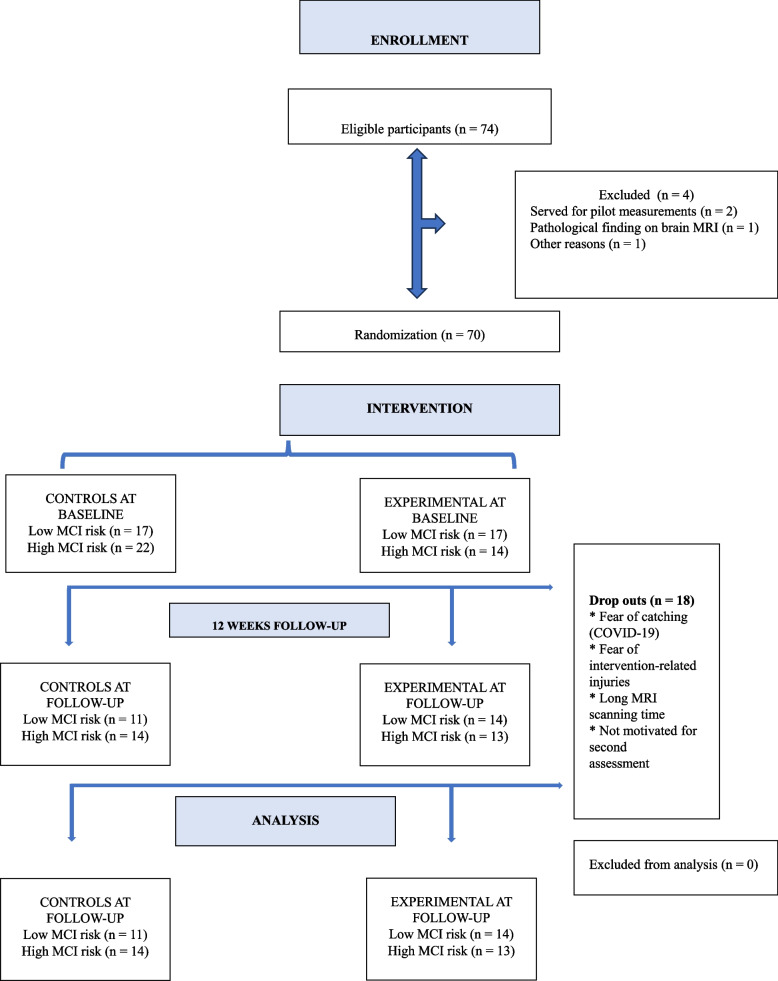
Participant flow diagram. Please note that some participants enrolled in this project were excluded or dropped-out because of MRI scanning, while no MRI results are presented in this paper. Abbreviations: MCI, Mild cognitive impairment; MRI: Magnetic resonance imaging

The experimental group underwent 12 weeks of resistance exercise training, while the control group underwent no intervention. Participants from both groups were instructed to continue their daily life routines as usual. Following a cognitive screening with the MoCA test in the Saules Family Medical Center in Kaunas, Lithuania, participants were invited twice on separate days for additional testing at the Lithuanian Sports University in Kaunas, Lithuania. For each participant, the same test conditions were provided at the same time of day (8 am to 11 am) before and after the 12 weeks period. All participants were instructed to avoid unusual physical activity, alcohol, and caffeine intake the day before testing and to sleep at least 7 h. They were asked to have breakfast at least 1–2 h before the experiment. During the first testing day, participants reported their demographic and medical characteristics (see Sect. " Demographic and medical characteristics"). All assessments (see Sect. " Physical activity assessment"-" Blood sampling") were performed before and after the 12 weeks intervention or control condition. See detailed description of the study procedure in Fig. 1 and participant flow diagram in Fig. 2.

### Demographic and medical characteristics

All participants completed a questionnaire battery that assessed their demographic and medical characteristics, such as age, sex, educational level, and smoking status. Educational levels were categorized as primary, secondary, or higher education.

### Physical activity assessment

Physical activity level was assessed using the IPAQ-SF. This self-report questionnaire comprises seven questions and four intensity levels of activity: 1) vigorous-intensity activity such as aerobics, 2) moderate-intensity activity such as leisure cycling, 3) walking, and 4) sitting [28]. Each activity type's frequency (days per week) and duration (minutes per day) in the last seven days are recorded. Each type of activity is characterized by METs (metabolic equivalent of task), and total IPAQ score is estimated by adding up the calculated MET-minutes within each physical activity intensity level (vigorous intensity = 8.0 MET, moderate intensity = 4.0, walking = 3.3 MET). Participants that indicated to burn less than 600 kcal/week are defined as sedentary, 600–3000 kcal/week as moderately physically active, and more than 3000 kcal/week as highly physically active.

### Body composition analysis

Body weight (in kg), height (in cm), body fat percentage (fat %), and body mass index (BMI, in kg/m^2^) were measured before and after the intervention. Weight and fat% were estimated using leg-to-leg bio-impedance analysis (BIA, Tanita TBF-300-A).

### Maximum voluntary knee extension force

The maximum voluntary contraction (MVC, in Newton meters) of isometric knee extension torque of the dominant leg was measured with Biodex System 3 dynamometer (Biodex Medical Systems, NY, USA). The highest MVC value out of two trials was recorded.

### Neurocognitive assessments

#### MoCA test

A MoCA examination was conducted by a mental health care professional to evaluate cognitive abilities. This test is considered reliable and consists of 12 items that assess seven cognitive domains, including visuospatial ability and executive functioning, naming, memory, attention, language, abstract reasoning, and orientation. All items contribute to a total score of up to 30 points, with a higher score indicating stronger cognitive functioning. One point is added to the total score if the participant had less than 13 years of education. The participants were classified into different groups based on their MoCA test scores, with a cutoff score of 26 being used for stratification. It is worth noting that a MoCA test score of 25 or lower is generally considered indicative of a high risk of MCI in an otherwise healthy geriatric population [22].

#### ANAM test

Specific cognitive domains were tested with four selected tests of the ANAM4 (Automated Neuropsychological Assessment Metrics, version 4) test battery. The ANAM4 test system consists of a library of 28 computer-based self-administered tests that assess different aspects of neurocognition including executive functions and attentional processes. Since maintaining attention, inhibitory control, basic computational skills, and working memory are crucial for independent daily living in older adults, we have selected specific subtests to evaluate these functions before and after the intervention. These subtests comprised two-choice reaction time, Go/No-Go, mathematical processing, and memory search tasks. Subjects completed the cognitive tests using a Lithuanian Sports University (LSU) computer running the test suite software in a quiet environment. The software automatically provides the already averaged results of each test. Outcome measure was the response time (in milliseconds, ms). Accuracy measures were used to exclude trials with more than 50% incorrect responses, as this may indicate that the subject did not understand the task. We decided also to delete response times that were faster than the best percentile of young male college students, based on normative values presented in the ANAM4 user manual, considering that this likely indicates that the participants did not adequately perform or understand the task and attained the 50% correct responses by chance [29]. However, none of the participants ‘ results had to be excluded based on this decision. A familiarization session took place 48-72 h before the testing day and on the testing day the participants were allowed one practice trial before the results were being recorded.

#### 2-choice reaction time test

We used this test to assess processing speed and alternating attention. It contains a motor speed component. The 2-choice reaction time test measures choice reaction time by presenting the participant with a "*" or "o" on the screen. The individual is instructed to respond as quickly as possible by pressing the left or right mouse button as soon as the stimulus appears.

#### Go/No-go test

It is used to assess response inhibition. The participant is presented with two characters, “o” and “x” and needs to respond as quickly as possible to the “x” character each time the stimulus appears. The subject is instructed to do nothing when the character “o” appears (inhibit response).

#### Memory search test

The results of the memory search test are used as an index of attention, immediate recognition, and verbal working memory. The program uses letters and symbols to assess verbal working memory as symbolic and non-verbal subparts. The user sees a positive memory set of four letters on the screen (e.g., “T B Q U”). Then, individual characters are displayed, and the participant needs to press mouse buttons to indicate if each character is or is not a member of the positive memory set.

#### Mathematical processing test

The mathematical processing test results are used as an index of concentration, working memory, and computational skills. During the test, the participant needs to solve an arithmetic problem (e.g., “4 + 8–5 = ”). The task involves only three single-digit numbers and two operators. The subject needs to indicate whether the answer is less or higher than five.

### Blood sampling

Serum IL-6, KYN, and IGF-1 concentrations were measured using the ELISA method (ELISA, Biotek, model ELX 800) with spectrophotometry (Spark 10 M, Tecan Group Ltd. Zürich, Switzerland) by an experienced technician. A nurse drew the venous blood samples from the antecubital vein into 5 mL EDTA-K3 vacuum tubes. All blood samples were collected between 9:00 a.m. and 1:00 p.m. The second blood collection was carried out 2 to 4 days after the last exercise session for participants in the experimental group. The tubes were gently inverted 8–10 times immediately after blood collection and kept at room temperature for no more than 30–35 min until centrifugation for 15 min at 4,000 g centrifugal force. Subsequently, serum was aliquoted into 1.5 mL polypropylene tubes. The serum samples were frozen immediately after serum separation and kept at -80 °C in the freezing room of the LSU Institute of Sports Science and Innovation laboratory until further examination.

The IL-6 ELISA kit was purchased from DIAsource ImmunoAssays S.A., Belgium (KAP1216). The lower detection limit is 2 pg/mL. The KYN ELISA kit was purchased from MyBiosource, Inc., USA. The lower detection limit is 45.7 ng/mL. The IGF-1 ELISA kit was purchased from IBL International, GMBH, Germany (MD58011). The lower detection limit is 0.03 ng/mL.

### Training intervention

RT intervention was conducted over 12 weeks in the Lithuanian Sport University gym in accordance with the National Strength and Conditioning Association (USA) position statement on resistance training for older adults [30]. Two to ten days prior to the exercise intervention, participants were familiarized with the RT procedure and underwent 1-repetition maximum (1RM) testing. Two resistance training sessions were scheduled per week with a minimum of two days apart. Warm-up consisted of 5-min cycling on a cycle ergometer at an intensity (in Watts) approximately equal to the participant’s body weight in kilograms, followed by a few dynamic stretching and activation exercises including lunges, butt kicks, side step lunges, half-squats, and front and side cross swings. The training program comprised four exercises, namely knee extension (1), incline leg press(2), hamstring curls (3), and calf raise (4), using resistance training equipment from Technogym (Italy).

Each exercise was performed for 2–3 sets of 6–10 repetitions, at 70–85% of the baseline 1RM, with a 2-min rest between sets and a 3-min rest between exercises. From week 1 to 3, participants did 8–10 repetitions, starting at 70–75% 1-RM; from week 4 to 9, they worked 6–8 repetitions, starting at 75–80% 1-RM; and from week 10 to 12, they did six repetitions starting at 80–85% 1-RM. After the first session and during each of the three training blocks the weight was adjusted according to the participants’ rate of perceived exertion (RPE) on a 10-point Borg scale. The weight was increased when the older adult indicated a score below seven on ten. The exercise sequence was periodically randomized. Qualified trainers supervised all training sessions.

### Statistical analysis

IBM SPSS Statistics version 27 (IBM Corp., Armonk, NY) was used to perform all analyses. Data were initially inspected for outliers and normality. Extreme outliers were defined as values lying more than 3 × the interquartile range away from the median and were excluded. Normality was checked graphically using PP-plots and histograms and numerically by a kurtosis and skewness measure between -2 and + 2. If the normality assumptions were not met, data was log transformed. Homoscedasticity was tested with the Levene’s test.

First, independent t-tests and Chi^2^ tests (or Fisher Exact tests, if the expected count in any of the cells was below 5) were used to assess group differences in baseline variables for continuous and categorical variables respectively. Two-way ANCOVA was used with either post-intervention blood biomarker levels or ANAM test results as a dependent variable. Group (experimental versus control) and cognitive status (low MCI risk versus high MCI risk) were entered as fixed factors, and age and pretest values of the dependent variable as covariates. Body fat % was entered as an additional covariate for analysis with IL-6 or KYN levels. We chose this approach instead of a three-way repeated measures ANOVA, because it was demonstrated that ANCOVA tests taking into account the pretest value of the dependent variable by entering it as a covariate in the model, rather than as a level of the time factor in repeated measures ANOVA, reduce the population error variance and are therefore more powerful and precise [31]. Bivariate correlation *R*-values were calculated between the pre- to post-intervention changes in blood biomarker levels and ANAM test results for total group and experimental group. Spearman’s rho was chosen, because of the non-normal distribution of some of the outcome measures. Statistical significance was accepted at α = 0.05.

## Results

### Participants characteristics

Eighteen of 70 participants (25.7%) dropped out during the intervention reporting the following drop-out reasons: COVID-19 infection or fear of getting infected, lack of motivation, or intervention related trauma or fear of injury. Participants’ ages ranged from 60 to 85 years (mean age: 69 ﻿ ± 6.2 years) and over half (54.3%) were women. The descriptive values of the baseline characteristics after excluding the drop-outs are presented in Table 1. There was a significant difference at baseline between the experimental and control groups in educational levels and kilocalories burned per week. Missing values existed for IL-6 (*n* = 3), KYN (*n* = 1) and IGF-1 (*n* = 1). The MVC of knee extension torque was measured only in a subgroup of participants (*n* = 31), used for analysis in a study of Sheoran et al. (2023) [25].

**Table 1:** Baseline participant characteristics and group differences

	Control (*n* = 25)	Experimental (*n* = 27)	Total (*n* = 52)	*p*-value
Age	69.0 (5.9)	70.7 (5.6)	69.9 (5.8)	0.293
Sex:				
- Male	12 (48.0%)	12 (44.4%)	24 (46.2%)	0.797
- Female	13 (52.0%)	15 (55.6%)	28 (53.8%)	
Education:				0.038*
- Higher	22 (88.0%)	20 (74.1%)	42 (80.8%)	
- Secondary	1 (4.0%)	7 (25.9%)	8 (15.4%)	
- Basic	2 (8.0%)	0 (0%)	2 (3.8%)	
Smoking status				1.000
- Smoker	1 (4.0%)	2 (7.4%)	3 (5.8%)	
IPAQ-SF kcal/week	5759.8 (4542.3)	3296.7 (2711.3)	4480.9 (3873.1)	0.024*
IPAQ-SF PA level:				0.116
- sedentary	1 (4.0%)	4 (14.8%)	5 (9.6%)	
- moderately active	8 (32.0%)	13 (48.1%)	21 (40.4%)	
- highly active	16 (64.0%)	10 (37.0%)	26 (50.0%)	
Height (cm)	168.6 (7.8)	165.0 (8.8)	166.9 (8.4)	0.080
Weight (kg)	77.4 (21.8)	77.3 (14.7)	77.4 (18.8)	0.988
BMI (kg/m^2^)	27.4 (3.3)	28.4 (4.6)	28.1 (5.0)	0.385
Body fat (%)	31.1 (8.0)	32.2 (9.6)	31.5 (9.4)	0.656
MVC (Nm)	158.1 (32.4)	141.6 (38.5)	149.0 (36.3)	0.212
MoCA score	24.5 (3.4)	25.6 (2.5)	25.0 (3.0)	0.204
High MCI risk	14 (56.0%)	13 (48.1%)	27 (51.9%)	0.571
ANAM test results				
- 2-choice reaction time (ms)	604.4 (113.3)	637.1 (123.9)	621.4 (118.9)	0.326
- Go/No-go (ms)	475.5 (56.2)	471.8 (57.8)	473.6 (56.5)	0.818
- Memory search (ms)	1193.1 (298.7)	1306.8 (239.6)	1252.1 (273.0)	0.135
-Mathematical processing (ms)	3342.5 (1104.3)	3170.0 (716.3)	3253.0 (918.2)	0.511
Blood biomarkers				
- IL-6 (pg/mL)	7.5 (8.0)	7.8 (8.8)	7.6 (8.4)	0.888
- KYN (ng/mL)	1550.1 (724.3)	1686.1 (872.3)	1619.4 (798.1)	0.548
- IGF-1 (ng/mL)	124.7 (50.0)	123.4 (62.0)	124.0 (55.9)	0.934

Continuous parameters are expressed as mean values (SD), *p*-values are derived from independent t-tests; categorical parameters are expressed as n (% of total), *p*-values are derived from Chi^2^ tests or Fisher Exact tests. Significant *p*-values are marked *

*Abbreviations*: *ANAM* Automated Neuropsychological Assessment Metrics, *BMI* Body mass index, *ELISA* Enzyme-linked immunosorbent assay, *IGF-1* Insulin-like growth factor-1, *IL-6* Interleukin-6, *IPAQ-SF* International Physical Activity Questionnaire-Short Form, *KYN* Kynurenine, *MCI* Mild cognitive impairment, *MoCA* Montreal Cognitive Assessment, *MVC* Maximum voluntary knee extension torque

### Blood biomarkers following 12 weeks of RT

Between exercise and control group effects and group x cognitive status interaction effects derived from the ANCOVA test were nonsignificant for IGF-1, IL-6, and KYN. Cognitive status significantly affected KYN level (*p* = 0.015); individuals with higher MCI risk had higher KYN levels.

Of note, effect size of the group effect for IL-6 was of a moderate level (_p_η^2^ = 0.078, *p* = 0.089). IL-6 levels decreased by 6.5% in the control group while they increased by 43.5% in the intervention group. This change was particularly evident among older persons with high MCI risk, with IL-6 change of + 53.7% in the exercise group and -8.9% in the control group. Table 2 presents the absolute values, and Table 4 contains the two-way ANCOVA results for the blood biomarkers.

**Table 2:** Pre- and post-intervention blood biomarker absolute outcome values and percentage change

	Group	N	Pre-Intervention	Post-Intervention	Δ (%)
IGF-1 (ng/mL)	EXP	24	119.0 (57.6)	139.2 (85.4)	+ 17.0
	-hrMCI	12	118.0 (55.2)	143.2 (81.2)	+ 21.4
	-lrMCI	12	120.1 (62.4)	135.3 (92.8)	+ 12.7
	CON	18	112.5 (50.2)	136.3 (66.4)	+ 21.2
	-hrMCI	8	117.7 (58.3)	141.3 (58.3)	+ 20.1
	-lrMCI	10	108.3 (45.5)	132.4 (75.1)	+ 22.2
	Total	42	116.2 (54.0)	138.0 (77.0)	+ 18.8
	-hrMCI	20	117.9 (54.9)	142.4 (71.2)	+ 20.8
	-lrMCI	22	114.7 (54.4)	133.9 (83.3)	+ 16.7
IL-6 (pg/mL)	EXP	23	8.5 (9.2)	12.2 (12.2)	+ 43.5
	-hrMCI	12	6.7 (5.5)	10.3 (11.2)	+ 53.7
	-lrMCI	11	10.5 (12.0)	14.4 (13.5)	+ 37.1
	CON	20	7.7 (8.2)	7.2 (8.9)	-6.5
	-hrMCI	10	4.5 (4.2)	4.1 (3.1)	-8.9
	-lrMCI	10	11.2 (10.2)	10.7 (11.8)	-4.5
	Total	43	8.1 (8.6)	9.9 (11.0)	+ 22.2
	-hrMCI	22	5.6 (4.9)	7.3 (8.8)	+ 30.4
	-lrMCI	21	10.8 (10.9)	12.7 (12.6)	+ 17.6
KYN (ng/mL)	EXP	23	1582.3 (755.7)	1301.3 (606.4)	-17.8
	-hrMCI	11	1434.8 (650.6)	1442.3 (579.5)	+ 0.5
	-lrMCI	12	1717.4 (846.1)	1172.2 (626.2)	+ 46.5
	CON	22	1616.4 (709.1)	1507.1 (578.2	-6.8
	-hrMCI	12	1308.2 (441.0)	1722.2 (593.8)	+ 31.6
	-lrMCI	10	2017.2 (808.8)	1227.5 (439.8)	-39.1
	Total	45	1599.3 (724.8)	1404.2 (595.0)	-12.2
	-hrMCI	23	1366.2 (538.2)	1593.9 (591.8)	+ 16.7
	-lrMCI	22	1853.7 (823.8)	1197.3 (537.7)	-35.4

Only the values of the participants with pre-and post-intervention measurements were used for analysis

*Abbreviations*: *CON* Control, *EXP* Experimental, *hrMCI* High risk for mild cognitive impairment, *IGF-1* Insulin-like growth factor-1, *IL-6* Interleukin-6, *KYN* Kynurenine, *lrMCI* Low risk for mild cognitive impairment

### Cognitive performance changes

ANCOVA results did not indicate a significant group or cognitive status effect on neurocognitive performance. However, there was a significant interaction effect of group and cognitive status on Go/No-go test score (*p* = 0.010). The absolute values showed that resistance exercise improved reaction time more in older adults with high MCI risk (-4.3%) compared to healthy ones (-0.9%). In contrast, in the control group reaction times increased in older adults with high MCI risk (+ 2.1%), but decreased in healthy older adults (-6.8). Table 3 presents the absolute values and Table 4 contains the two-way ANCOVA results for cognitive tests.

**Table 3:** Pre- and post-intervention ANAM outcome values and percentage change

	Group	N	Pre-Intervention	Post-Intervention	Δ (%)
ANAM 2-choice reaction time (ms)	EXP	27	637.1 (123.9)	637.0 (133.1)	-0.0
	-hrMCI	13	662.7 (124.0)	645.0 (133.5)	-2.7
	-lrMCI	14	613.4 (123.4)	629.7 (137.3)	+ 2.7
	CON	24	599.3 (116.4)	593.2 (98.2)	-1.0
	-hrMCI	13	579.4 (123.0)	616.7 (113.9)	+ 6.4
	-lrMCI	11	621.0 (110.4)	567.6 (74.7)	-8.6
	Total	51	619.8 (120.8)	616.9 (119.3)	-0.5
	-hrMCI	26	622.7 (128.2)	631.4 (122.7)	+ 1.4
	-lrMCI	25	616.8 (115.5)	602.4 (116.3)	-2.3
ANAM Go/No-go (ms)	EXP	27	471.8 (57.8)	459.8 (62.6)	-2.5
	-hrMCI	13	480.2 (56.1)	459.6 (56.7)	-4.3
	-lrMCI	14	464.1 (60.3)	460.0 (69.8)	-0.9
	CON	23	472.3 (55.6)	463.3 (71.6)	-1.9
	-hrMCI	12	476.2 (53.0)	488.3 (76.0)	+ 2.1
	-lrMCI	11	468.0 (60.5)	436.0 (58.0)	-6.8
	Total	50	472.0 (56.2)	461.4 (66.2)	-2.2
	-hrMCI	25	478.2 (53.6)	473.3 (66.8)	-1.0
	-lrMCI	25	465.8 (59.1)	449.4 (64.7)	-3.5
ANAM Memory search (ms)	EXP	27	1306.8 (239.6)	1328.3 (316.3)	+ 1.6
	-hrMCI	13	1326.3 (212.7)	1288.8 (266.9)	-2.8
	-lrMCI	14	1288.7 (268.8)	1365.0 (362.4)	+ 5.9
	CON	23	1161.7 (271.5)	1204.9 (360.7)	+ 3.7
	-hrMCI	12	1145.9 (208.4)	1259.3 (408.3)	+ 9.9
	-lrMCI	11	1178.9 (337.3)	1145.5 (309.0)	-2.8
	Total	50	1240.0 (262.4)	1271.5 (339.7)	+ 2.5
	-hrMCI	25	1239.7 (225.8)	1274.6 (335.0)	+ 2.8
	-lrMCI	25	1240.4 (299.4)	1268.4 (351.1)	+ 2.3
ANAM Mathematical processing (ms)	EXP	27	3170.0 (716.3)	3187.6 (704.1)	+ 0.6
	-hrMCI	13	3286.8 (919.7)	3226.4 (609.3)	-5.7
	-lrMCI	14	3061.6 (467.9)	3151.6 (803.6)	+ 2.9
	CON	23	3267.5 (1109.8)	3068.3 (1110.9)	-6.1
	-hrMCI	12	3521.2 (1355.6)	3320.2 (1376.6)	-5.7
	-lrMCI	11	2990.8 (725.7)	2793.5 (686.4)	-6.6
	Total	50	3214.9 (909.7)	3132.7 (905.9)	-2.6
	-hrMCI	25	3399.3 (1131.1)	3271.4 (1027.8)	-3.8
	-lrMCI	25	3030.5 (582.5)	2994.0 (760.9)	-1.2

Only the values of the participants with pre-and post-intervention measurements were used for analysis

*Abbreviations*: *ANAM* Automated Neuropsychological Assessment Metrics, *CON* Control, *EXP* Experimental, *hrMCI* High risk for mild cognitive impairment, *lrMCL* Low risk for mild cognitive impairment

**Table 4:** Two-way ANCOVA for blood biomarker and ANAM test results

	Group		Cognitive status		Group*Cognitive status	
	*p*-value	_p_η^2^	*p*-value	_p_η^2^	*p*-value	_p_η^2^
IGF-1	0.439	0.016	0.612	0.007	0.717	0.004
IL-6	0.089	0.078	0.401	0.020	0.476	0.014
KYN	0.372	0.021	0.015*	0.147	0.328	0.025
ANAM 2-choice reaction time	0.389	0.017	0.374	0.018	0.272	0.027
ANAM Go/No-Go	0.674	0.004	0.285	0.026	0.010*	0.141
ANAM Memory search	0.610	0.006	0.925	0.000	0.089	0.064
ANAM Mathematical Processing	0.175	0.041	0.736	0.003	0.752	0.002

The dependent variables are presented in the first row. *p*-values and effect sizes (partial eta squared) are given for the group effect, the cognitive status effect and their interaction (Group*Cognitive status). Significant *p*-values are marked *. The ANCOVA was adjusted for the dependent variable’s baseline value and for age. The general linear model for IL-6 and KYN were additionally adjusted for fat%. Of note, pre-intervention KYN, post-intervention IGF-1, post-intervention IL-6, pre-intervention ANAM mathematical processing, post-intervention ANAM memory search and post-intervention ANAM mathematical processing were log-transformed due to a non-normal distribution, and in pre-intervention IL-6 and post-intervention IL-6 and KYN an extreme outlier was removed before analysis

*Abbreviations*: *ANAM* Automated Neuropsychological Assessment Metrics, *IGF-1* Insulin-like growth factor-1, *IL-6* Interleukin-6, *KYN* Kynurenine, *MCI* Mild cognitive impairment, _*p*_*η*^*2*^ Partial eta squared

### Correlations between blood and cognitive changes

**Fig. 3:**
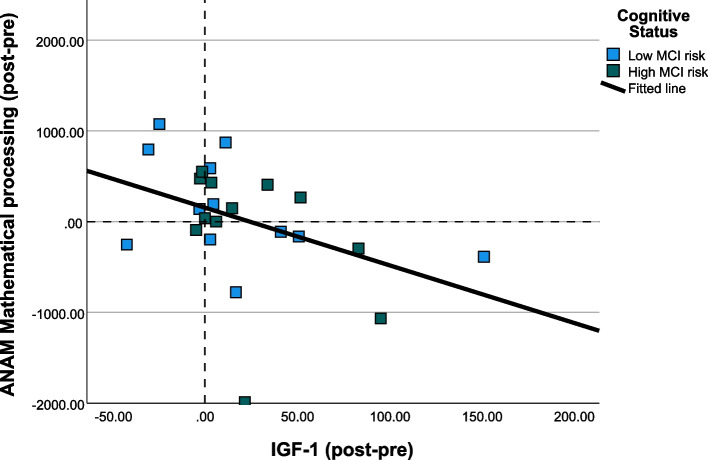
Bivariate relationship between pre-to-post changes in IGF-1 and changes in ANAM Mathematical processing response time (ms) in experimental group. Positive values mark increases from pre- to post test. Abbreviations: ANAM, Automated Neuropsychological Assessment Metrics; IL-6, interleukin-6; MCI, mild cognitive impairment

A significant negative correlation between changes in IGF-1 levels and changes in mathematical processing response time in the exercise group (r = -0.497, *p* = 0.014) was found (see Table 5, Fig. 3). Furthermore, there was a significant negative correlation between changes in IL-6 level and changes in memory search score when combining experimental and control group (*r* = -0.313, *p* = 0.038) (see Additional file 1). Of note, after Bonferroni correction the needed significance levels was α = 0.002 (i.e. α = 0.05/21 significance tests) to which none of the significant results comply, suggesting we cannot state with certainty that these findings are robust.

**Table 5:** Bivariate correlations between changes in blood biomarkers and cognition in experimental group

		ΔIL-6	ΔKYN	ΔANAM 2-choice reaction time	ΔANAM Go/No-go	ΔANAM Memory search	ΔANAM Mathematical processing
ΔIGF-1	R	-0.271	0.022	-0.025	0.276	-0.001	-0.497*
	*p*	0.210	0.922	0.907	0.192	0.997	**0.014**
ΔIL-6	R		0.155	-0.121	0.125	-0.289	0.254
	*p*		0.492	0.581	0.569	0.181	0.242
ΔKYN	R			-0.004	0.004	-0.128	-0.167
	*p*			0.986	0.986	0.559	0.446
ΔANAM 2-choice reaction time	R				0.432*	0.158	0.209
	*p*				**0.025**	0.431	0.296
ΔANAM Go/No-go	R					0.105	-0.206
	*p*					0.602	0.303
ΔANAM Memory search	R						-0.156
	*p*						0.436

Significant values are marked in bold, significance level * *p* < 0.05

Δ values were calculated by subtracting the post-intervention value from the pre-intervention value

Spearman’s rho correlation values are presented. Significant correlations are marked in bold

*Abbreviations*: *ANAM* Automated Neuropsychological Assessment Metrics, *IGF-1* Insulin-like growth factor-1, *IL-6* Interleukin-6; KYN, kynurenine

## Discussion

Our research provides additional insights into the effects of resistance exercise on inflammatory and neurotrophic blood biomarkers and cognitive performance in older adults with low or high risk of MCI. In addition, we obtained new findings on the relationship between exercise-induced changes in circulating biomarkers and cognitive performance.

Our primary finding was that larger increases in IGF-1 levels following RT were associated with larger improvements in response time on the mathematical processing task. Overall, IGF-1 increased both in the RT and control group, with no significant differences between groups. Notably, although the relationship between IGF-1 changes and cognitive performance changes is in line with findings from previous literature (see below), our result did not reach the Bonferroni corrected significance level. These results can be compared to those of Tsai and colleagues. In 2015 and 2019 respectively, they reported associations between changes in IGF-1 levels and changes in response time on a variant of the oddball task following a 12 month RT intervention in healthy older adults [32], but not with changes in performance and a switching task following a 16 week RT intervention in older adults with MCI [33]. Furthermore, a cross-sectional study by Al-Delaimy and colleagues showed that IGF-1 levels relate significantly to verbal fluency and global cognition in older men [17]. IGF-1 is thought to have neuroprotective effects, promoting the growth and survival of neurons in the brain, as well as reducing inflammation and oxidative stress [34]. Furthermore, it has been reported that either injecting IGF-1 or exercise-induced IGF-1 increases can improve the transcription of hippocampal Brain-Derived Neurotrophic Factor (BDNF) [35, 36], widely known as a mediator of exercise-induced cognitive improvement [37]. These findings support the hypothesis that IGF-1 may play a role in RT-induced cognitive benefits but cannot claim it acts as a mediator of the exercise-cognition effect. For mediation analysis, a larger sample size is needed.

Furthermore, we discovered that increases over time in IL-6 levels were associated with improvements in memory search scores when evaluating the total group of participants. This was in contrast to our hypothesis and previous studies that have demonstrated an inverse association between working memory and IL-6 levels in older adults [38, 39]. However, in the RT group, the relationship between changes in IL-6 and memory search response time was no longer significant. At the molecular level, the link between IL-6 and memory function is previously explained by cytokines' involvement in synaptogenesis, neurogenesis, and memory consolidation [40]. Since the hippocampus has the highest expression of inflammatory cytokine receptors for IL-6 [41], peripheral IL-6 change may affect hippocampus-related memory score. It should be noted, however, that IL-6 has both pro- and anti-inflammatory actions [42] and pro-inflammatory cytokines can both be beneficial and detrimental for neuroplasticity depending on their cerebral concentration [43].

Another important finding from our study is the significant ANCOVA interaction effect for group x cognitive status on Go/No-go response time. This finding indicates that the post-intervention response time on the Go/No-go test differs between RT and control group taking into account the baseline Go/No-go results depending on the older adults cognitive status. In line with our hypothesis, RT enhanced the improvement over time on this inhibition task to a higher extent in older adults with high MCI risk compared to healthy older adults. In turn, it is likely to interpret that the supportive effect of exercise is more pronounced in older adults with more cognitive loss. This finding is conform that of a meta-analysis on the effect of aerobic exercise, that indicated a larger effect size for improvements in cognition in participants with MCI compared to healthy and demented participants [13]. Furthermore, the fact that this was only found for the response inhibition task, and not for the processing speed and two working memory tasks is in line with previous meta-analyses suggesting that executive functions are more likely to respond to resistance exercise, with the working memory component of executive functions being less responsive [11, 44]. Finally, it is important to take into consideration that the changes in inhibitory control observed in the RT group for high risk MCI participants could be partly due to social interaction resulting from group activities. Studies have shown that participating in social leisure activities can help older adults maintain their cognitive abilities [45]. Similarly, engaging in group activities has been linked to increased cognitive function by promoting an overall sense of well-being [46]. Therefore, future studies should recruit control groups engaged in social interaction to reduce the potential confounding factors related to social experiences.

Finally, we found that post-intervention KYN levels were significantly higher in older adults with high MCI risk compared to older adults with low MCI risk. Consistent with our finding, cognitive impairment has been associated with higher KYN levels in individuals with type 2 diabetes mellitus [47] and acute post COVID-19 individuals [48]. KYN levels increase in case of elevated pro-inflammatory cytokine concentrations, which stimulates its conversion from tryptophan by indoleamine-2,3-dioxygenase [49]. It has previously been related to neuroinflammation [23], neurodegeneration [23], cognitive decline [50] and increased dementia risk [51]. It should be noted that KYN can be metabolized within the brain to quinolinic acid and kynurenic acid. The former was found to have detrimental effects on neuroplasticity by inducing neuroinflammation and an overactivation of NMDA receptors, while the latter was found to be an antagonist of the NMDA receptor with beneficial effects in low concentrations, but detrimental effects in high concentrations [52–54]. All these findings suggest that KYN may be a marker of neurodegeneration and cognitive decline.

A limitation of the study is that it took place during the COVID-19 pandemic. Three participants dropped-out because of confirmed COVID-19, while others may have had subclinical infections. We did not exclude participants with a history of COVID-19 before inclusion in the study. As COVID-19 has been reported to induce molecular signatures of aging in the brain [55], we should not overlook that our blood biomarker and cognitive findings may have interfered with this condition.

To conclude, this randomized controlled trial indicated that 12 weeks of resistance exercise did not significantly affect peripheral biomarkers in older adults with low or high MCI risk. However, when taking into account the older adults cognitive status, RT positively affected inhibitory control, particularly in older adults with a high risk of MCI. Moreover, our study results suggest that RT-induced increases in the neurotrophic factor IGF-1 may play a role in RT-induced improvements in mathematical processing. Finally, KYN is put forward as a potential blood biomarker related to cognitive impairment.

### Supplementary Information

**Additional file 1: Supplementary Table 1.** Bivariate correlations between changes in blood biomarkers and cognition in both experimental and control group.** Supplementary Fig. 1.** Bivariate relationship between pre-to-post changes in IL-6 and ANAM Memory search response time (ms) in both experimental and control group.

## Data Availability

The datasets used and/or analysed during the current study are available from the corresponding author on reasonable request.

## Abbreviations

ANAM: Automated Neuropsychological Assessment Metrics
BMI: Body mass index
ELISA: Enzyme-linked immunosorbent assay
IGF-1: Insulin-like growth factor-1
IL-6: Interleukin-6
IPAQ-SF: International Physical Activity Questionnaire-Short Form
KYN: Kynurenine
MCI: Mild cognitive impairment
MoCA: Montreal Cognitive Assessment
MRI: Magnetic resonance imaging
RPE: Rate of perceived exertion
RT: Resistance training
