# Supplementary material for: Resistance training's impact on blood biomarkers and cognitive function in older adults with low and high risk of mild cognitive impairment: a randomized controlled trial
Source: Eur Rev Aging Phys Act. 2024 Apr 10;21:9. doi: 10.1186/s11556-024-00344-9 (PMC11005144; doi:10.1186/s11556-024-00344-9)
Supplement: Supplementary file 1 — Additional file 1: Supplementary Table 1. Bivariate correlations between changes in blood biomarkers and cognition in both experimental and control group. Supplementary Fig. 1. Bivariate relationship between pre-to-post changes in IL-6 and ANAM Memory search response time (ms) in both experimental and control group. [file 11556_2024_344_MOESM1_ESM.docx]

| **Supplementary table 1.** Bivariate correlations between changes in blood biomarkers and cognition in both experimental and control group | | | | | | | |
| --- | --- | --- | --- | --- | --- | --- | --- |
|  |  | ΔIL-6 | ΔKYN | ΔANAM 2-choice reaction time | ΔANAM Go/No-go | ΔANAM Memory search | ΔANAM Mathematical processing |
| ΔIGF-1 | R | -0.148 | -0.018 | -0.018 | 0.163 | -0.105 | -0.125 |
|  | *p* | 0.370 | 0.910 | 0.908 | 0.307 | 0.514 | 0.435 |
| ΔIL-6 | R |  | -0.063 | -0.294 | -0.040 | **-0.313*** | -0.005 |
|  | *p* |  | 0.686 | 0.053 | 0.798 | **0.038*** | 0.975 |
| ΔKYN | R |  |  | 0.105 | 0.260 | 0.085 | -0.016 |
|  | *p* |  |  | 0.486 | 0.084 | 0.579 | 0.916 |
| ΔANAM  2-choice reaction time | R |  |  |  | **0.407**** | 0.135 | 0.208 |
|  | *p* |  |  |  | **0.003**** | 0.350 | 0.147 |
| ΔANAM Go/No-go | R |  |  |  |  | 0.132 | 0.089 |
|  | *p* |  |  |  |  | 0.937 | 0.537 |
| ΔANAM Memory search | R |  |  |  |  |  | -0.011 |
|  | *p* |  |  |  |  |  | 0.937 |
| Significant values are marked in bold, significance level * *p* < 0.05, ** *p* < 0.01.  Δ values were calculated by subtracting the post-intervention value from the pre-intervention value.  Spearman’s rho correlation values are presented. Significant correlations are marked in bold.  Abbreviations: ANAM, Automated Neuropsychological Assessment Metrics; IGF-1, insulin-like growth factor-1; IL-6, interleukin-6; KYN, kynurenine | | | | | | | |


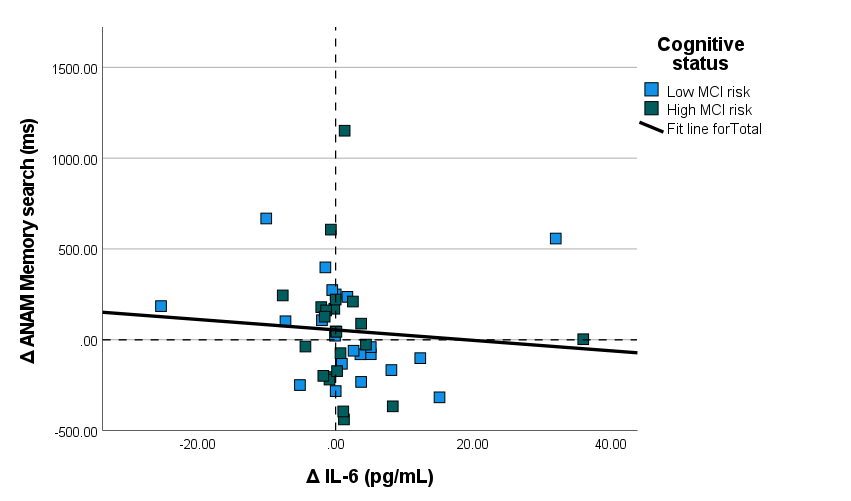


**Supplementary Figure 1.** Bivariate relationship between pre-to-post changes in IL-6 and ANAM Memory search response time (ms) in both experimental and control group. Positive values mark increases from pre- to post test. Abbreviations: ANAM, Automated Neuropsychological Assessment Metrics; IL-6, interleukin-6; MCI, mild cognitive impairment.
